# The National Pension Fund for Nurses

**Published:** 1888-02-18

**Authors:** 


### The National Pension Fund for Nurses.

The first meeting of the Council was held at the office, 38, Old Jewry, on Friday, the 10th inst. Mr. Walter H. Burns (Messrs. J. S. Morgan and Co.) was appointed chairman, and Mr. Burdett deputy-chairman of the Council. Mr. Thomas Bryant (senior surgeon to Guy's Hospital), Mr. R. Brudenell Carter (the St. George's and National Hospitals), and Major Ross, M.P. (chairman of the Middlesex Hospital), were elected members of the Council. The tables, rates of premium, and other forms which the actuary had prepared, were submitted, and arrangements were made for the completion of the necessary prospectuses, etc., for the information and guidance of those waiting to join the Fund. The Earl of Aberdeen, Lord Rothschild, Messrs. E. A. Hambro, Henry Hucks Gibbs, and Junius S. Morgan were elected vice-presidents; and the Countess of Rosebery and Lady Rothschild patronesses. It was reported that several hundred names of those anxious to join the Fund had been registered during the last three weeks, in addition to the 1,400 names previously announced. Mr. Philip Grove was appointed secretary, and all names and inquiries should be addressed to him at the offices of the Fund, 38, Old Jewry, E.C.

Works of which the effect is to correct our errors, to strengthen our reason, to elevate our spirit, to improve our mind, are as productive in their way as those that tend to lower the price of meat or bread.—*Edmond About.*

